# Pembrolizumab输注过程中出现低血压：病例报告并文献综述

**DOI:** 10.3779/j.issn.1009-3419.2019.11.09

**Published:** 2019-11-20

**Authors:** 鹏 宋, 晓彤 张, 力 张

**Affiliations:** 100730 北京，中国医学科学院，北京协和医学院北京协和医院呼吸内科 Department of Respiratory Medicine, Peking Union Medical College Hospital, Chinese Academy of Medical Science and Peking Union Medical College, Beijing 100730, China

**Keywords:** Pembrolizumab, 输注反应, 低血压, Pembrolizumab, Infusion reaction, Hypotension

## Abstract

免疫检查点抑制剂在中国获批时间较短，真实世界的临床数据尚处于收集阶段，国内程序细胞死亡蛋白1（programmed death-1, PD-1）治疗相关不良反应的报道罕见。笔者报道1例Pembrolizumab输注过程中出现低血压，血压恢复后成功输注的案例，希望能为免疫检查点抑制剂的应用提供参考，为患者提供最大的临床获益。

## Abstract (en)

Immunological checkpoint inhibitors have been approved for a short period of time in China, and real-world clinical data are still in the collection stage. Reports of domestic programmed death-1 (PD-1) treatment-related adverse reactions are rare. The author reported a case of hypotension in the process of Pembrolizumab infusion and successful infusion after blood pressure recovery, hoping to provide reference for the application of immunological checkpoint inhibitors, to provide patients with the greatest clinical benefit.

近年来，免疫治疗成为继手术、放疗、化疗后又一种重要的抗肿瘤手段。其中抗程序细胞死亡蛋白1（programmed death-1, PD-1）及配体（PD-1 ligand, PD-L1）免疫检查点阻断剂，在肿瘤治疗中取得了突破性进展。随着PD-1/PD-L1阻断剂在临床上逐步推广，越来越多的免疫相关副反应（immune-related adverse events, irAEs）引起关注。本文对PD-1/PD-L1阻断剂治疗引起的输注反应以及临床处理方法进行讨论。

## 1 临床资料

患者男性，68岁。身高167 cm，体质量50 kg。2017年1月查体时胸部计算机断层扫描（computed tomography, CT）发现右肺下叶占位性病变；正电子发射型计算机断层显像（positron emission computed tomography, PET）-CT提示右肺下叶占位伴脱氧葡萄糖（fluoro-2-deoxy-D-glucose, FDG）代谢异常增高，纵膈及双肺门淋巴结代谢增高。2017年3月行电视辅助胸腔镜手术（video-assisted thoracic surgery, VATS）右下肺切除及淋巴结清扫，术后病理提示肺中-低分化鳞癌。术后给与GP方案（吉西他滨和顺铂联合用药）化疗4个疗程。此后规律复查。2019年2月患者因左侧肢体麻木无力就诊，头部磁共振成像（magnetic resonance imaging, MRI）提示右侧脑部巨大占位；PET-CT提示双肺门及纵隔淋巴结代谢增高，考虑术后复发。完善组织PD-L1检测为5%。脑部转移灶给与全脑放疗，因患者体质偏弱，经充分沟通后，内科治疗方案为白蛋白紫杉醇400 mg联合Pembrolizumab 200 mg静脉输注。除外化疗及免疫治疗禁忌证后，2019年8月7日14时20分开始Pembrolizumab静脉输注，输注前生命体征平稳，血压130 mmHg/80 mmHg。输注Pembrolizumab 20 min后，14时40分患者心电监护仪提示血压85 mmHg/50 mmHg，立即床旁水银柱血压计左右臂各复测1次，左臂血压82 mmHg/50 mmHg，右臂血压80 mmHg/55 mmHg。其余生命体征平稳。患者无头晕、黑朦等不适，四肢暖，皮肤无花斑，该患者无高血压、心血管疾病等慢性病史，在Pembrolizumab输注前并没有使用其他任何辅助药物。考虑低血压与Pembrolizumab所致输液反应相关，立即停止Pembrolizumab输注，予生理盐水1, 000 mL快速补液，补液完毕后血压可恢复至100 mmHg/70 mmHg，尿量无减少。与患者充分沟通后，17点给予白蛋白紫杉醇输注，过程顺利，无不适。查询Pembrolizumab说明书后，已配置好的溶液可低温冰箱保存24 h，因此当天没有重新输注Pembrolizumab。8月8日9时，患者复测血压为120 mmHg/80 mmHg，向患者充分沟通继续输注风险后，其对可能的风险表示知情，并要求输注剩余的Pembrolizumab。9时30分给予剩余Pembrolizumab输注，10时30分输注完毕。复测血压125 mmHg/70 mmHg。过程顺利，未诉不适。21 d以后患者进行了第2次白蛋白紫杉醇联合Pembrolizumab输注，输注过程顺利，无不良反应发生。

## 2 讨论

这是国内首例报道Pembrolizumab输注过程中出现低血压的病例，并且血压恢复正常后成功完成了剩余输注。这可以纳入ICIs所致的输注反应，其可能表现出一些固定的症状，如发热、僵硬、瘙痒、低血压、呼吸困难、胸部不适、皮疹、荨麻疹、血管性水肿、喘息或心动过速，也包括需要紧急处理的过敏或超敏反应。

目前上市的PD-1/PD-L1抑制剂中，各种药物发生输注反应的比例不一。接受Avelumab治疗的患者中，输注反应（所有级别）的发生率约为15%-17%；而其他ICIs治疗输注反应的发生率多低于5%（表 1）^[1-11]^。因Avelumab是唯一一个强ADCC活性的PD-1/PD-L1抗体，其余PD-1/PD-L1抗体均采用弱ADCC活性的IgG4亚型，或者糖基化改造弱化ADCC活性的IgG1亚型。因此这一原因可能导致接受Avelumab治疗的患者中输注反应的发生率明显高于其他ICIs。免疫治疗联合化疗会增加输注反应的复杂性。在Keynote-047研究中，Pembrolizumab联合化疗组和化疗组相比3度-4度的输注反应发生率明显提高（2.4% *vs* 0.6%）^[12]^。这提示临床医生在免疫治疗联合化疗越来越普遍使用的情况下，应高度警惕输注反应的发生，准确识别并及时处理。

**1:** 与免疫检查点抑制剂有关的输注反应 Infusion reactions related to immunological checkpoint inhibitors

No.	Type of cancer	PD-1/PD-L1	Infusion reaction		References
			Level 1-2	Level 3-4	
1	Malignant pleural mesothelioma	Pembrolizumab	4.0%		Alley 2017
2	Merkel cell carcinoma	Avalumab	17.0%		Kaufman 2016
3	NSCLC	Durvalumab	1.0%	1.0%	Garassino 2018
4	NSCLC	Avalumab	15.0%	1.5%	Barlesi 2018
5	NSCLC	Nivolumab	5.7%		Hida 2017
6	NSCLC	Nivolumab	3.9%		Yamazaki 2016
7	Gastric cancer	Pembrolizumab	2.0%		Shitara 2018
8	Cervical cancer	Pembrolizumab	3.1%		Chung 2019
9	NSCLC	Pembrolizumab	1.0%	< 1.0%	Mok 2019
10	Melanoma	Pembrolizumab	4.8%		Nishio 2016
11	Hodgkin's lymphoma	Nivolumab	5.9%		Maruyama 2017
NSCLC: non-small cell lung cancer; PD-1: programmed death-1; PD-L1: PD-1 ligand.					

目前并没有相关文献探讨免疫治疗输注反应的发生机制。但免疫治疗也是一种液体的输注，因此推断发生机制应该等同于普通输注反应的发生机制。可能与药物纯度、溶液种类、输液环境、患者体质及基础疾病等有关。但免疫治疗有其特殊性，需要进一步探讨其可能输注反应的机制。

中国临床肿瘤协会（Chinese Society of Clinical Oncology, CSCO）免疫毒性管理指南推荐对于轻微或中度的输注反应需要对症治疗、减慢输液速度或暂停输液。对于再次发生输注反应的、危及生命的3级-4级输注反应应永久停药^[13]^。笔者认为本例患者继续输注Pembrolizumab无不良后果值得临床医生借鉴，对于仅有单一症状的输注反应，可在症状完全好转后密切监视患者情况，对可能产生的后果需向患者充分交待，得到知情同意后继续输注Pembrolizumab，这可使患者获得最大的临床获益。

## References

[1] Alley EW, Lopez J, Santoro A (2017). Clinical safety and activity of pembrolizumab in patients with malignant pleural mesothelioma (KEYNOTE-028): preliminary results from a non-randomised, open-label, phase 1b trial. Lancet Oncol.

[2] Kaufman HL, Russell J, Hamid O (2016). Avelumab in patients with chemotherapy-refractory metastatic Merkel cell carcinoma: a multicentre, single-group, open-label, phase 2 trial. Lancet Oncol.

[3] Garassino MC, Cho BC, Kim JH (2018). Durvalumab as third-line or later treatment for advanced non-small-cell lung cancer (ATLANTIC): an open-label, single-arm, phase 2 study. Lancet Oncol.

[4] Barlesi F, Vansteenkiste J, Spigel D (2018). Avelumab versus docetaxel in patients with platinum-treated advanced non-small-cell lung cancer (JAVELIN Lung 200): an open-label, randomised, phase 3 study. Lancet Oncol.

[5] Hida T, Nishio M, Nogami N (2017). Efficacy and safety of nivolumab in Japanese patients with advanced or recurrent squamous non-small cell lung cancer. Cancer Sci.

[6] Yamazaki N, Takenouchi T, Fujimoto M (2017). Phase 1b study of pembrolizumab (MK-3475; anti-PD-1 monoclonal antibody) in Japanese patients with advanced melanoma (KEYNOTE-041). Cancer Chemother Pharmacol.

[7] Shitara K, Ozguroglu M, Bang YJ (2018). Pembrolizumab versus paclitaxel for previously treated, advanced gastric or gastro-oesophageal junction cancer (KEYNOTE-061): a randomised, open-label, controlled, phase 3 trial. Lancet.

[8] Chung HC, Ros W, Delord JP (2019). Efficacy and safety of pembrolizumab in previously treated advanced cervical cancer: results from the phase Ⅱ KEYNOTE-158 study. J Clin Oncol.

[9] Mok TS, Wu YL, Kudaba I (2019). Pembrolizumab versus chemotherapy for previously untreated, PD-L1-expressing, locally advanced or metastatic non-small-cell lung cancer (KEYNOTE-042): a randomised, open-label, controlled, phase 3 trial. Lancet.

[10] Nishio M, Hida T, Atagi S (2016). Multicentre phase Ⅱ study of nivolumab in Japanese patients with advanced or recurrent non-squamous non-small cell lung cancer. ESMO Open.

[11] Maruyama D, Hatake K, Kinoshita T (2017). Multicenter phase Ⅱ study of nivolumab in Japanese patients with relapsed or refractory classical Hodgkin lymphoma. Cancer Sci.

[12] Paz-Ares L, Luft A, Vicente D (2018). Pembrolizumab plus chemotherapy for squamous non-small-cell lung cancer. N EngI J Med.

[b13] 13Immunotherapy-related thrombocytopenia management guidelines. In: Working Committee of the Chinese Society of Clinical Oncology Guidelines. ed. Guide to toxicity management related to immunological checkpoint inhibitors. 1^st^ ed. Beijing: People Health Publishing House, 2019. 53.免疫治疗相关血小板减少管理指南.见: 中国临床肿瘤学会指南工作委员会主编.免疫检查点抑制剂相关的毒性管理指南.第1版.北京: 人民卫生出版社, 2019. 53.

